# The Effect of Parent Psychological Distress on Child Hyperactivity/Inattention During the COVID-19 Lockdown: Testing the Mediation of Parent Verbal Hostility and Child Emotional Symptoms

**DOI:** 10.3389/fpsyg.2020.567052

**Published:** 2020-12-10

**Authors:** Daniela Marchetti, Lilybeth Fontanesi, Serena Di Giandomenico, Cristina Mazza, Paolo Roma, Maria Cristina Verrocchio

**Affiliations:** ^1^ Department of Psychological, Health and Territorial Sciences, G. d’Annunzio University of Chieti-Pescara, Chieti, Italy; ^2^ Department of Humanities, University of Urbino Carlo Bo, Urbino, Italy; ^3^ Department of Neuroscience, Imaging and Clinical Sciences, G. d’Annunzio University of Chieti-Pescara, Chieti, Italy; ^4^ Department of Human Neuroscience, Faculty of Medicine and Dentistry, Sapienza University of Rome, Rome, Italy

**Keywords:** parent psychological distress, verbal hostility, emotional symptoms, hyperactivity and attention, COVID- 19

## Abstract

The coronavirus disease 2019 (COVID-19) health crisis is strongly affecting the psychological well-being of the general population. According to a very recent literature, the imposed lockdown and social distancing measures have generated a series of negative outcomes, including fear of the future, anxiety, and somatization symptoms. Few studies have investigated the impact of the COVID-19 pandemic on the well-being of parents and children, and still fewer studies have assessed the relationship between the psychological health of parents and children. The present study aimed at understanding the effect of parents’ psychological distress and verbal aggression on behavioral and emotional symptoms of children during the COVID-19 lockdown. Using an online survey administered in the first weeks of the lockdown in Italy, we explored the mediating effects of parent verbal hostility and child emotional symptoms on the relationship between parent distress and child hyperactivity/inattention in a sample of 878 Italian parents (87.4% mothers; mean_age_ = 40.58). Two hypotheses were proposed: (1) parent distress would significantly predict child hyperactivity/inattention, and (2) parent verbal hostility and child emotional symptoms would mediate the association between parent distress and child hyperactivity/inattention. The serial mediated model confirmed both hypotheses, suggesting that higher rates of psychological distress in parents were associated with higher levels of hyperactivity/inattention in children. Parent verbal hostility and child emotional problems were also found to positively mediate this relation. Our results may be used to improve sociopsychological interventions in the general population in the near future. They may also contribute to the clinical definition of therapeutic paths for parents and families.

## Introduction

The coronavirus disease 2019 (COVID-19) health crisis is strongly affecting the psychological well-being of the general population. To prevent the spread of the virus, governments worldwide have imposed social distancing measures, closed schools, and enforced mandatory lockdowns, forcing individuals to deal with new and challenging situations (Brodeur et al., 2020). In Italy, a series of restrictions of increasing severity began on February 23, 2020, with a regional lockdown initiated in Lombardy (northern Italy), where the country’s first case of COVID-19 was registered (Lazzerini and Putoto, 2020). Gradually, the measures became more stringent, culminating in a national lockdown on March 11, involving the closure of schools and prohibitions on general activities.

While many studies have assessed the impact of the pandemic on the general population (Mazza et al., 2020), few studies have assessed the effects of the lockdown on the parent-child relationship or parent and child well-being (Brown et al., 2020; Gassman-Pines et al., 2020; Griffith, 2020; Marchetti et al., 2020; Patrick et al., 2020). In addition to generating negative effects in the general population, the COVID-19 lockdown may also be creating a particularly stressful environment for parents, who may face concerns over their family’s health, their children’s isolation from teachers and peers, and their management of homeschooling and daily commitments (e.g., working remotely and meeting financial obligations; Fontanesi et al., 2020; Romero et al., 2020). Furthermore, although very few children have been infected with COVID-19 in Italy, children are not immune to the tragic impact of the pandemic, but may experience fear, isolation, uncertainty, worry, irritability, and inattention (Jiao et al., 2020).

Several studies have documented the damaging effects of psychological stress in children following negative events; such effects include drastic changes in emotional and behavioral patterns and sleep and eating habits, higher levels of anxiety and depression, and impaired social interactions (Hoven et al., 2005; Klein et al., 2009; Lai et al., 2015; Verrocchio et al., 2018). As suggested by the literature, these symptoms may be partly determined by the direct effect of experiencing a negative event; however, parents’ mental health and parenting style behaviors may also play a key role in influencing children’s adjustment during stressful situations (Pfefferbaum et al., 2015, 2016).

Parents’ general mental health and psychological distress are well-established risk factors for psychological problems in children (Siegenthaler et al., 2012; Verrocchio et al., 2013; Patrick et al., 2020). The literature shows that maternal mental health is associated with poor behavioral, emotional, social, and cognitive outcomes in children (Glasheen et al., 2010; Verrocchio, 2016), whereas paternal depressive symptomatology contributes to negative emotional and behavioral outcomes in children (Weitzman et al., 2011). Overall, psychological distress has been found to be associated with adverse behavioral and emotional outcomes in children (Verrocchio et al., 2019); in particular – and regardless of parent gender – parents’ mental health has been found to relate to emotional symptoms in younger children and hyperactive behavior in children of all ages (Amrock and Weitzman, 2014).

Parenting style can be described as a constellation of practices toward children that create an emotional environment and influence child development and well-being. The role of parenting style behaviors on children’s emotional and behavioral problems is widely cited in the literature (Rinaldi and Howe, 2012; Braza et al., 2015). In particular, maternal verbal hostility has been shown to be responsible for children’s negative emotional arousal and internalizing symptoms (Smarius et al., 2019; Pozzi et al., 2020), and parental verbal aggression (i.e., yelling and bursts of rage) has been found to be associated with depression and anxiety symptoms in children and preadolescents (Möller et al., 2016). Furthermore, although parenting style tends to be relatively stable, some parenting style behaviors can be heightened or triggered by parents’ compromised psychological well-being (Tavassolie et al., 2016), especially during stressful situations (Miki et al., 2019) such as the COVID-19 lockdown.

Aggressive maternal and paternal parenting behaviors have been shown to result in emotional problems in children, and these emotional problems may trigger the onset of inattention and hyperactive/impulsive symptoms (Eisenberg et al., 2001). Symptoms of emotional distress in children (e.g., irritability, sadness, and worry) are frequently accompanied by externalizing behaviors (e.g., restlessness, temper tantrums, and inability to concentrate) and may even predict attention-deficit/hyperactivity disorder (ADHD) symptoms over time (Brocki et al., 2019). In fact, recent studies have suggested that emotional problems may positively predict inattention symptoms and that high levels of physical (e.g., headaches and stomachaches) and internalizing symptoms – associated with emotional distress – are typically present before a diagnosis of ADHD (Han et al., 2020). Both internalizing and externalizing symptoms in children and preadolescents can have serious consequences for their interpersonal, cognitive, and psychological domains, such as impaired social competency, substance abuse, poor academic performance, and decreased mental health (Creavey et al., 2018; Gargano et al., 2018).

In light of these findings, it is reasonable to suggest that the present critical and unexpected situation of emergency may increase parents’ mental distress; this may be reflected in a verbally aggressive parenting style, which may negatively influence children’s psychological well-being. Understanding this relation and the outcomes is essential for properly addressing the needs of parents and children in the near future and for developing new interventions to help people cope with traumatic events. Italy was not only one of the first – and most severely affected – countries to suffer from the COVID-19 pandemic, but it is also subject to frequent natural disasters, earthquakes, and floods, resulting in displaced families who are forced to live in shelters and to reorganize their lives accordingly, with dramatic consequences for children’s general well-being.

Although a wide range of maladaptive parenting practices may contribute to an undesirable parent-child relationship, the current study focused on the role of a single component of aggressive behavior in parents. Specifically, the present research aimed at understanding the relationship between parent psychological distress, parent verbal hostility, and child behavioral and emotional symptoms during the COVID-19 lockdown. Although there is evidence of a bidirectional relation between parents and children, with children’s characteristics eliciting certain parenting practices (e.g., Zadeh et al., 2010; Pearl et al., 2014), some studies have demonstrated that parental practices affect children’s behavior much more strongly than the reverse (Choe et al., 2013). On this basis, we explored the mediating effects of parent verbal hostility and child emotional symptoms on the relationship between parent psychological distress and child hyperactivity/inattention in a sample of Italian parents. Drawing on the process-oriented model of developmental trajectories and child adjustment (Cummings et al., 2000; Miragoli and Verrocchio, 2008), which suggests that different factors and environments may encourage development along an adaptive or potentially maladaptive trajectory, we proposed two hypotheses: (1) parent psychological distress would significantly predict child hyperactivity/inattention behavior and (2) parent verbal hostility and child emotional symptoms would mediate the association between parent psychological distress and child hyperactivity/inattention behavior.

## Materials and Methods

### Participants and Procedure

The study sample was part of a wider research project investigating the effect of the COVID-19 pandemic on the mental health of Italian parents and children. From April 3 to 14, participants completed an anonymous online survey on the Qualtrics platform, after reading and approving a consent form describing the aims of the study, participant rights, and the data treatment procedure. The survey took approximately 20 min to complete. Participants were randomly recruited through social media and snowball sampling and selected according to the following inclusion criteria: (a) being at least 18 years old and (b) being a parent to at least one child aged 3–13 years, with whom they were spending the lockdown. With respect to the latter criterion, we selected this age range for the children because we expected that the parents of these children would be experiencing a higher education-related burden during the lockdown, as younger children often require more parental assistance in their lessons and homework than do adolescents. The research protocol was approved in accordance with the Declaration of Helsinki and its revisions (General Assembly of the World Medical Association, 2014) by the local ethics committee (Board of the Department of Human Neuroscience, Faculty of Medicine and Dentistry, Sapienza University of Rome, n. 6.2020).

### Measures

The survey consisted of, first, a set of sociodemographic questions investigating parents’ age, gender, marital status, work status, and level of education and the age and gender of the target child. Following this, the survey presented a series of standardized measures of parent psychological distress and verbal hostility and child emotional symptoms and hyperactivity-inattention behavior during the COVID-19 lockdown.

Psychological distress of parents was assessed with the General Health Questionnaire-12 (Piccinelli et al., 1993; Piccinelli and Politi, 1993; Giorgi et al., 2014). This is a 12-item measure of somatic symptoms, depression, anxiety, insomnia, and social dysfunction. Participants were asked to evaluate how their distress had changed since the beginning of the lockdown on a scale from 0 to 3, with higher scores indicating a worse mental health condition (example item: “Have you felt constantly under strain?”). In the present sample, internal consistency was good (*α* = 0.85).

Verbal hostility of parents was assessed using three items (yells or shouts when child misbehaves, argues with child, and explodes in anger toward child) of the Italian short version of the Parenting Styles and Dimensions Questionnaire (Robinson et al., 1995, 2001; Confalonieri et al., 2009). Each item was rated on a five-point Likert scale ranging from 1 (*never*) to 5 (*always*). Participants were asked to evaluate the number of times they had used verbal hostility toward their child since the beginning of the lockdown. Total scores were created by summing the three-item scores. In this study, the Verbal Hostility subscale had acceptable internal consistency (*α* = 0.76).

Emotional symptoms and hyperactivity/inattention of children were assessed by parents using the Italian version of the Strengths and Difficulties Questionnaire-Parent Report (SDQ; Goodman, 2001; Marzocchi et al., 2002). The SDQ is a widely used brief behavioral screening instrument that assesses children’s positive and negative attributes across five scales, each composed of five items: Emotional Symptoms, Conduct Problems, Hyperactivity/Inattention, Peer Problems, and Prosocial Behavior. For the present study, the Emotional Symptoms and Hyperactivity/Inattention subscales were used to assess emotion and behavioral problems. Participants were asked to evaluate – on a three-point Likert scale ranging from 0 (*not true*) to 2 (*certainly true*) – the presence of emotional and behavioral problems in their child during the lockdown. Example items of the two subscales used in this study include “Restless, overactive, and cannot stay still for long” and “Often unhappy, downhearted, or tearful.” In the current study, the Emotional Symptoms and Hyperactivity/Inattention subscales had acceptable internal consistency (*α* = 0.76 and *α* = 0.71, respectively).

### Data Analysis

Prior to the main analysis, we examined the data using frequencies and descriptive statistics. Data were screened for deviation from parametric assumptions and met the requirements without transformation. To test our hypotheses, we used Pearson correlations to investigate associations between parent psychological distress, parent verbal hostility, child emotional symptoms, and child hyperactivity-inattention experienced during the COVID-19 lockdown. Following this, we evaluated the association between child gender, age, and hyperactivity/inattention behavior in order to determine whether to include covariates in the hypothesis-testing model. Finally, we employed PROCESS Model 6 in SPSS 26.0 to examine Hypotheses 1 and 2. In addition, a 95% bias-corrected confidence interval with 5,000 bootstrap samples was applied to determine the significance of the mediational effect.

## Results

### Descriptive Statistics and Correlations

A total of 878 caregivers (87.4% mothers; mean_age_ = 40.58 ± 6.41) who responded to the survey met the inclusion criteria and were included in the study. Descriptive data on the sample are summarized in Table 1. Means and standard deviations of all variables and the correlations between variables are displayed in Table 2. The results demonstrated significant and positive correlations between parent psychological distress during the COVD-19 lockdown, parent verbal hostility, child emotional symptoms, and child hyperactivity-inattention behavior, providing preliminary support for our hypotheses. Child gender and age were significantly related to hyperactivity/inattention behavior. Specifically, being male and younger was associated with higher hyperactivity/inattention behavior.

**Table 1:** Descriptive characteristics of the sample (*n* = 878).

	Total sample
Parental role, n (%)	
Mother	767 (87.4)
Father	111 (12.6)
Age	
Mean (SD)	40.58 (6.41)
Range	23–67
Marital status, n (%)	
Single	32 (3.6)
Married	643 (73.2)
Living with a partner	127 (14.5)
Separated/divorced	72 (8.2)
Widowed	4 (0.5)
Work status, n (%)	
Employed	738 (84.1)
Unemployed	140 (15.9)
Educational level, n (%)	
Less than high school	55 (6.2)
High school	343 (39.1)
More than high school	480 (54.6)
Geographic area, n (%)	
North	222 (25.3)
Center	277 (31.5)
South	379 (43.2)
Child gender, n (%)	
Male	451 (51.7)
Female	427 (48.3)
Child age	
Mean (SD)	7.54 (3.16)
Range	3–13

**Table 2:** Means, standard deviations, and correlations between study variables.

Variable	Mean	SD	1	2	3	4	5
Child gender^a^	–	–	–				
Child age	7.54	3.16	0.01	–			
Child hyperactivity/inattention	3.20	2.34	−014^**^	−0.15^**^	–		
Parent psychological distress	19.37	5.93	0.03	−0.04	0.20^**^	–	
Parent verbal hostility	7.74	2.38	−0.07^*^	0.04	0.39^**^	0.24^**^	–
Child emotional symptoms	2.03	1.95	0.01	0.06	0.33^**^	0.27^**^	0.30^**^

a Point-biserial coefficient.

* *p* < 0.05;

** *p* < 0.01.

### Serial Mediation Model

**Figure 1:**
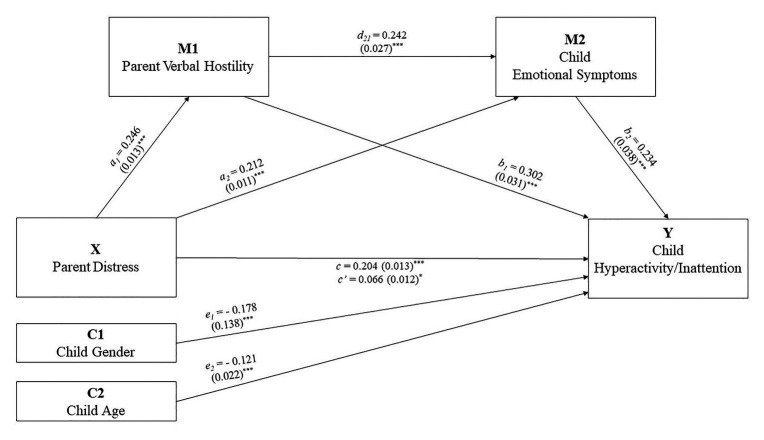
Serial multiple mediation model. Numbers represent standardized coefficients. Numbers within parentheses are standardized errors. ^*^ *p* < 0.05, ^***^ *p* < 0.001.

There was a statistically significant direct effect confirming Hypothesis 1, that parent psychological distress during the COVID-19 lockdown would predict child hyperactivity-inattention behavior. Furthermore, indirect effects were also statistically significant, providing support for Hypothesis 2 (Table 3; Figure 1), that parent verbal hostility and child emotional problems during the lockdown would be positive serial mediators of the relationship between parent psychological distress and child hyperactivity-inattention.

**Table 3:** Model coefficients for the serial mediation analysis.

	Nonstandardized coefficients (SE/boot SE)	Bootstrapping BC 95% CI		Standardized coefficients (SE/boot SE)	Std bootstrapping BC 95% CI		*p*
*R* ^2^ = 0.2512, *F*(5.867) = 58.167, *p* < 0.001		Lower	Upper		Lower	Upper	
Total effect	0.081 (0.013)	0.056	0.106				<0.001
Direct effect	0.026 (0.012)	0.002	0.050				0.033
Indirect effects							
Total indirect effect	0.055 (0.008)	0.041	0.070	0.138 (0.019)	0.103	0.175	
*a*_1_ *b*_1_	0.029 (0.005)	0.019	0.040	0.074 (0.013)	0.049	0.101	
*a*_2_ *b*_2_	0.020 (0.005)	0.012	0.029	0.050 (0.011)	0.030	0.073	
*a*_1_ *d*_21_ *b*_2_	0.006 (0.001)	0.003	0.009	0.014 (0.003)	0.008	0.021	

## Discussion

The present study examined the links between parent psychological distress, parent verbal hostility, and child emotional symptoms and hyperactivity-inattention during the COVID-19 lockdown, with an emphasis on the identification of potential mediating processes among these variables.

Throughout the pandemic, the psychological condition of parents and children has been an area of professional and institutional concern, worldwide. Nonetheless, despite the significant clinical interest in this topic, little effort has been devoted to its study. The present research thus extends our empirical knowledge of the relationship between parents’ mental health and children’s psychological well-being during a pandemic.

A primary goal was to explore the associations between parents’ psychological distress and children’s hyperactivity/inattention during the COVID-19 lockdown. The results showed that parents’ psychological distress significantly predicted children’s hyperactivity/inattention. This result was consistent with previous studies confirming that parents’ psychological distress is a risk factor for the development of externalizing problems in children (e.g., Glasheen et al., 2010; Siegenthaler et al., 2012; Amrock and Weitzman, 2014). Furthermore, as expected on the basis of previous studies in the general population (e.g., Mayes et al., 2020), children’s gender and age were significantly related to hyperactivity/inattention. Specifically, male gender and younger age were associated with higher hyperactivity/inattention. It is likely that the COVID-19 lockdown may be particularly stressful for parents, who may be facing concerns about the economic and physical health of their family; their children’s isolation from peers and teachers; and the management, duration, and outcomes of their homeschooling (Fontanesi et al., 2020; Schmidt et al., 2020). These feelings and concerns may cause psychological distress, which is an emotional state characterized by depressive and anxious symptoms. Parents experiencing high psychological distress may be less attentive to and warm with their children. They may also transfer the burden of their emotional distress to their children, which could affect their children’s adjustment. These results are in line with recent research suggesting that parental distress, a disadvantaged economic situation following the COVID-19 lockdown (i.e., loss of job or income), and social isolation represent important risk factors for child abuse and neglect, family violence, and a deterioration of the parent-child relationship (Brown et al., 2020; Gassman-Pines et al., 2020; Patrick et al., 2020).

The second aim of our study was to test parent verbal hostility and child emotional symptoms as mediators of the association between parent psychological distress and child hyperactivity/inattention. While the association between parent and child mental health in community samples is well-established (e.g., Smith, 2004; Goodman et al., 2011), many authors have argued the need to study the mechanisms through which the psychological symptoms of parents and their children are associated (Powdthavee and Vignoles, 2008; Baiocco et al., 2019). Various processes – not yet fully understood – may be used to explain the association between parent psychological distress and child behavioral problems. Parenting practices have long been cited as an important risk factor for child externalizing problems (Prevatt, 2003; Marmorstein and Iacono, 2004). Specifically, retrospective studies have demonstrated that verbal abuse during childhood is related to externalizing and internalizing disorders in adulthood, such as mood and anxiety disorders, eating disorders, substance abuse disorders, personality disorders, and schizophrenia, as well as to suicide risk (Carr et al., 2013; Falgares et al., 2018). In a study of children aged 9–12 years, Donovan and Brassard (2011) showed that maternal verbal aggression was associated with depressive symptoms, delinquency, peer overt and relational victimization, and low self-esteem.

The results of the present study show that parent verbal hostility and child emotional problems during the COVID-19 lockdown were positive serial mediators of the relationship between parent psychological distress and child hyperactivity/inattention. The association between parent verbal hostility and child externalizing symptoms is consistent with the findings of previous studies (Pinquart, 2017); however, the present study also considered the interrelation between child emotional symptoms and hyperactivity/inattention, due to a substantial lack of evidence on this topic. Understanding the co-occurrence and temporal dynamics of hyperactivity/inattention and emotional symptoms may be significant for explaining the development of hyperactivity/inattention from childhood through adolescence and into adulthood. In addition, a closer exploration of these processes is warranted because the additional presence of emotional symptoms can significantly affect quality of life, academic performance, adult adjustment, and lifetime psychiatric comorbidities (Wehmeier et al., 2010; Seymour et al., 2012; Verrocchio et al., 2015). Our findings show that emotional symptoms of children significantly predicted their hyperactivity/inattention. Previous studies have confirmed that hyperactive/impulsive symptoms may be related to negative emotionality, irritability, a low frustration tolerance, and conduct problems (Martel and Nigg, 2006; Sobanski et al., 2010; Lin and Gau, 2017). Furthermore, our results are consistent with the findings of a study in which children whose parents frequently expressed negative affect and low warmth displayed underregulated emotion and were more prone to displaying externalizing behaviors, relative to children whose parents were warm and frequently expressed positive affect (Eisenberg et al., 2001).

The findings of the current study should be interpreted in light of a balanced consideration of the limitations and strengths of the research. No data on the effects of parents’ psychological distress on children during the COVID-19 epidemic were available at the time of investigation. The use of a cross-sectional online survey enabled us to recruit as wide and representative a sample of the Italian population as possible, and it was considered the best way to obtain a timely picture of the national situation. However, the strengths of our study (i.e., the contribution to the knowledge base, the large sample size, and the use of validated psychological measures) should be measured against the study’s limitations, which include the cross-sectional study design, which prevented us from detecting the direction of causality; the exclusive reliance on parent-report data on children; the possibility of social desirability bias; the sample restriction to only those participants with Internet access; participants’ motivation to take the online assessment; and the low number of fathers enrolled.

Further longitudinal research in different countries involved in the COVID-19 pandemic and the use of observational measures and/or other informants of child emotional symptoms and behavior are needed. Future research efforts should continue to explore the negative influences of parents’ psychological distress during the COVID-19 lockdown on the well-being of children. For example, researchers could explore possible negative outcomes associated with specific types of parenting styles. Child development studies have demonstrated that warmth/hostility and restrictiveness/permissiveness are reliably related to child behavior, with a combination of high warmth/care and a moderate level of control providing the healthiest emotional and social outcomes (Burns and Dunlop, 1998). Research could also explore the individual and combined influence of parent psychological distress and parenting styles in the household, as well as the influence of specific parents’ occupation (e.g., nurse or doctor). Another important question is whether certain children are more or less vulnerable, depending on their personal characteristics and temperament, as well as previous psychopathological diseases that can contribute to the development of specific vulnerabilities.

The knowledge contributed by the present study about the influence of parents’ psychological distress on children’s well-being during a pandemic may have two notable practical implications. First, our research could have relevant implications in the social care setting and, particularly, in the implementation of population-based projects aimed at reducing parents’ psychological load. Second, the results are a useful starting point for identifying the aspects that can influence the parent-child relationship and children’s distress and for directing interventions in the context of family therapy.

## Data Availability Statement

The raw data supporting the conclusions of this article will be made available by the authors, without undue reservation.

## Ethics Statement

The studies involving human participants were reviewed and approved by Board of the Department of Human Neuroscience, Faculty of Medicine and Dentistry, Sapienza University of Rome (id nr. 6.2020). The patients/participants provided their written informed consent to participate in this study.

## Author Contributions

MV, DM, and PR contributed to the concept and the design of the research project. LF, MV, and DM contributed to the analysis of the literature. CM and SG contributed to the administration of online survey and acquisition of data. DM and LF contributed to the analysis, interpretation and writing of data. MV, DM, LF, PR, SG, and CM participated in drafting the article and revising it critically for important intellectual content. All authors contributed to the article and approved the submitted version.

### Conflict of Interest

The authors declare that the research was conducted in the absence of any commercial or financial relationships that could be construed as a potential conflict of interest.
